# Journal Impact Factors and High Aspirations of JMA Journal

**DOI:** 10.31662/jmaj.2024-0106

**Published:** 2024-06-28

**Authors:** Tsuguya Fukui

**Affiliations:** 1 Editor-in-Chief, JMA Journal

**Keywords:** JMA Journal, Journal Impact factor, Artificial Intelligence

The most recent issue of Journal Citation Reports has conferred Journal Impact Factor 1.5 to the JMA Journal. We are now at a stage in which the influence of our journal on the medical community can be objectively measured. Regarding the journal impact factor, we should bear in mind the fact that the impact factor only reflects the frequency a journal has been cited in recent years and not quality per se, like peer review process, caliber of reported content, or significance of findings ^(1)^. Nevertheless, this is an important step forward after the publication of 21 issues since the inaugural one in September 2018. It is truly my honor to have this moment in my sincere gratitude to Dr. Yutaka Atomi, my co-Editor-in-Chief until September 2023, for his devoted contribution to this Journal. Without his contributions, we could not have come to this stage.

The JMA Journal is an official medical journal in English under the auspices of the Japan Medical Association (JMA) and the Japanese Association of Medical Sciences (JAMS). It is characterized by an open access, high standard general medical journal carrying original, and review articles covering a wide range of clinical practice, basic sciences and news of medicine, medical care, and health policy, among others ^(2)^. Thanks to a slew of leaders and senior researchers in a variety of medical fields from around the world to form the entirety of editorial board of the JMA Journal, we could rapidly process articles submitted from all over the world and publish high-quality articles without delay. All along the line, as shown in the Table 1, the yearly numbers of submitted articles have been increasing, from 54 in 2018 to 202 in 2023, with a resultant increase in published ones, from 12 in 2018 to 105 in 2023. The number of articles submitted from outside Japan has also increased from 1 in 2018 to 21 in 2023.

**Table 1.** Number of Submitted and Published Articles.

Year	2018	2019	2020	2021	2022	2023	2024
Number of submitted articles	54	71	97	200	196	202	86
Number of published articles	12	34	51	74	95	105	57
Number of article submitted from abroad	1	9	17	25	30	21	7

The specialty areas mostly covered by submitted articles in the past include, roughly in descending order in 2023, Public Health, General Internal Medicine/Primary Care, Medical Education, Infectious Diseases, Cardiovascular Diseases, and Obstetrics and Gynecology, except for IT/CT, as shown in Table 2. They reflect important topics in current medical settings worldwide. Until now, IT/ICT has constituted only a small proportion. However, it is quite certain that this area gains exponential importance in basic and clinical research. As described in the Editorial “Integration of Artificial Intelligence (AI) in Medicines” by Dr. Masaki Mori, an editorial member of the JMA Journal, in this issue, AI has transformed numerous sectors, including medicine, with the recent enhancement in machine learning using neural networks and has the potential to enable the handling of information beyond the understanding of humans ^(3)^. We invite submissions of AI articles from all over the world.

**Table 2.** Proportion of Specialty Areas Covered by Submitted Articles (Yearly %).

Year	2018	2019	2020	2021	2022	2023	2024
Public Health	7.4	9.9	10.3	8.5	11.2	13.9	19.8
General Internal Medicine/Primary Care	0	0	5.2	5.5	9.7	13.9	19.8
Medical Education	0	1.4	0	4	3.6	8.4	7
Infectious Diseases	0	9.9	8.2	14	8.7	8.4	4.7
Cardiovascular Medicine	0	0	6.2	3.5	4.1	4	3.5
Obstetrics and Gynecology	1.9	1.4	4.1	6	8.2	3.5	3.5
IT/ICT	0	0	0	0	0.5	1	3.5

As expressed in the inaugural issue, we have high aspirations to be a leading medical journal from Japan for general readers from all over the globe. With journal impact factor having been conferred, we are now at the stage with measurable indicators to determine the degree of achievements of our aspirations. We are determined to make every effort to see the JMA Journal serve as a pivotal source of trustworthy and up-to-date information from medical communities in Japan and other countries as, the JAMA from the United States of America and the BMJ from the United Kingdom.

## Article Information

### Conflicts of Interest

None

### Disclaimer

Tsuguya Fukui is the Editor in Chief of JMA Journal and on the journal’s Editorial Staff. He was not involved in the editorial evaluation or decision to accept this article for publication at all.
